# Following the Francis report: investigating patient experience of mental health in-patient care

**DOI:** 10.1192/bjp.bp.115.171124

**Published:** 2016-07

**Authors:** E. Csipke, P. Williams, D. Rose, L. Koeser, P. McCrone, T. Wykes, T. Craig

**Affiliations:** E. Csipke, PhD, Division of Psychiatry, University College London, London; **P. Williams**, BSc, Health Services and Population Research, Institute of Psychiatry, Psychology and Neuroscience, King's College London, London; **D. Rose**, PhD, Service User Research Enterprise, Health Services and Population Research, Institute of Psychiatry, Psychology and Neuroscience, King's College London, London; **L. Koeser**, BSc, **P. McCrone**, PhD, Health Services and Population Research, Institute of Psychiatry, Psychology and Neuroscience, King's College London, London; **T. Wykes**, PhD, Department of Psychology, Institute of Psychiatry, Psychology and Neuroscience, King's College London, London; **T. Craig**, PhD, Health Services and Population Research, Institute of Psychiatry, Psychology and Neuroscience, King's College London, London, UK

## Abstract

**Background**

The Francis report highlights perceptions of care that are affected by different factors including ward structures.

**Aims**

To assess patient and staff perceptions of psychiatric in-patient wards over time.

**Method**

Patient and staff perceptions of in-patient psychiatric wards were assessed over 18 months. We also investigated whether the type of ward or service structure affected these perceptions. We included triage and routine care. The goal was to include at least 50% of eligible patients and staff.

**Results**

The most dramatic change was a significant deterioration in all experiences over the courseof the study. Systems of care or specific wards did not affect patient experience but staff were more dissatisfied in the triage system.

**Conclusions**

This is the first report of deterioration in perceptions of the therapeutic in-patient environment that has been captured in a rigorous way. It may reflect contemporaneous experiences across the National Health Service of budget reductions and increased throughput. The ward systems we investigated did not improve patient experience and triage may have been detrimental to staff.

The recent UK Francis report^1^ highlights in-patient experience as a central issue for health services. In acute mental health, studies often present a bleak picture, with in-patient care characterised as non-therapeutic, overcrowded, inefficient and poorly organised leading to high levels of stress for staff and a poor patient experience.^2–5^ But most of these studies were cross-sectional and with little rigour and have not investigated whether there are effects of different ward structures or admission policies. Neither have they taken a longitudinal perspective. This study reports on both of these issues and investigates whether there have been changes in the perceptions of care and whether different types of wards or services can mitigate these negative perceptions. We chose as our different ward structures a ‘triage’ ward system and more traditional in-patient care. Triage systems were proposed as a solution to bed overoccupancy, which can drive poor patient experiences. They provide a single admission ward for immediate but brief intensive treatment over 7–10 days with other wards providing longer-term targeted treatment for those continuing to require in-patient care.^6^ From a recent evaluation we know that these systems do not significantly reduce overall length of hospital stay^7^ but it may be that they can contribute to an outcome that is just as important – patient experience.

There is also, of course, a balance to be struck between what is good for patients and what is a reasonable working environment for staff. The high turnover on a triage ward may produce disadvantages to staff who need to rise to the challenges this system presents. This paper fills the evaluation gap by assessing both patient and staff perceptions of in-patient wards by comparing them over a relatively long time and between systems. The longer period (18 months) is vital as it enables the effects, particularly for staff of dealing with an increased patient turnover, to be assessed. We were also interested in whether one system is associated with a frequently reported benefit to patients – greater patient/staff interaction as this seems to be related to more satisfaction with services and whether more staff interaction is more costly. These sorts of studies produce a more nuanced approach that can provide evidence for recommendations about how to improve the patients' experience.

## Method

### Design

Patients and staff perceptions of care were collected on four occasions at 6-month intervals from all willing participants from all in-patient wards in two locations in the same large mental health trust; one operating a triage system and the other a routine care comparison site. We compared the systems as a whole and then investigated different ward types (triage ward, triage locality wards and routine care wards) to detect specific effects of individual ward types.

### Models of care

#### Triage service

This triage ward accepts all admissions and patients remain for a maximum of 7 days. At the end of this time, patients who require a longer hospital stay are transferred to one of three longer-term ‘locality’ wards. All wards are housed in a single building with mixed gender and an average of 18 beds.

#### Comparison service (routine care)

Patients are admitted to any of five wards where they remain for their in-patient stay. Wards had an average of 18 beds (two were mixed gender, one female and two male, and one ward specialised in first-episode psychosis). Initially wards were on three sites, but during the study consolidated onto one site.

### Participants

To ensure a broad range of opinions that represent general views, we aimed to recruit at least 50% of eligible staff and patients.

#### Patients

Inclusion criteria were: resident for >6 days, can communicate in English and provide informed consent. However, for the participants on the triage ward the minimum was 3 days to capture those with very short admissions. There were no diagnostic exclusion criteria. We interviewed patients only once in the study.

#### Nursing staff

Inclusion criteria were: permanent nursing staff at the time of recruitment or temporary staff if they had completed at least seven shifts in the previous month.

### Procedures

Bexley and Greenwich Research Ethics Committee granted approval (Ref: 07/H0809/49). Assessments were collected in one or two sessions from patients and one session from staff and took place at baseline (week 0), phase 1 (week 26), phase 2 (week 52) and phase 3 (week 78). Data were collected between November 2008 and August 2010.

### Measures

#### Main outcomes

**Patient views** To assess patient views we used Views on Inpatient Care (VOICE),^8^ an easy-to-understand and complete, 19-item self-report measure with good validity and reliability. A high score indicates a more negative perception.

**Staff views** To assess staff views we used Views of the Therapeutic Environment (VOTE),^9^ a 20-item self-report measure capturing perceptions of the daily pressures of working in acute mental health wards. Reliability and validity are good. A high score indicates worse perceptions.

#### Secondary and context measures

**Patients** We collected demographic and clinical information including age, gender, marital status, ethnicity and education. We also administered the Service Satisfaction Scale: Residential services evaluation (SSS-Res);^10^ higher scores indicate less satisfaction with services. The self-report questionnaire, Client Services Receipt Inventory – Inpatient (CITRINE),^11^ was used to record activity data on the in-patient ward and the time spent with health professionals perceived to be meaningful by the patient. In combination with data on the unit cost of staff time the cost of such perceived meaningful contacts can be calculated.^12^

We assessed patient functioning using two measures. The Global Assessment of Functioning (GAF),^13^ a 100-point researcher-rated scale based on observation, interview and medical records. High scores indicate better functioning. Researchers were trained by experienced raters (T.C. and E.C.) to produce reliable ratings. The Nurses Observational Scale for Inpatient Evaluation (NOSIE)^14^ is a nurse-completed 12-item scale focused on the assessment of socially unacceptable/unusual behaviour. High scores indicate worse behaviour.

**Staff** We collected demographic data including age, gender, ethnicity, grade, length of employment and education. We used the Maslach Burnout Inventory – Human Services Survey (MBI),^15^ a 22-item self-report scale with good psychometric properties, to assess for work-related ‘burnout’. High scores indicate worse burnout. We also used the Index of Work Satisfaction (IWS),^16^ a 44-item scale measuring health professionals' job satisfaction. High scores indicate worse satisfaction.

### Analysis

Four questions were investigated.

- Do perceptions of staff or patients change over time and if so can we explain it?
- Are patients' perceptions of the therapeutic environment (VOICE) and their satisfaction with services (SSS-RES) different between the two systems?
- Are staff perceptions of the therapeutic environment (VOTE), staff burnout (MBI) and work satisfaction (IWS) different between the two systems?
- What is the perceived care received and its cost (CITRINE) and does it differ between the two systems?

Patient outcomes, service use and its costs were modelled using linear regression in models that included time (data-collection phase) as a covariate. Sensitivity analyses using mixed-effects regression models took into account the clustering of patients by ward. In the cost regression, Huber-White standard errors were calculated to allow for non-normality of residuals. We accounted for the proportion of patients admitted to each ward during the study period and, based on a previous exploratory analysis, we adjusted for patients' age, education status, ethnicity and previous admission.

As some staff members were interviewed more than once, a random-effects regression was fitted but otherwise the models were the same as those underlying the patient analyses.

We tested whether our length of stay exclusion criteria affected the results by excluding triage system patients admitted for less than 7 days and no results changed.

We compared different ward types (triage ward, longer stay ward or routine acute ward) by estimating specific contrasts and carried out these analyses with and without controlling for patient functioning. We investigated predictors of patient and staff perceptions of the therapeutic environment using multiple regression models to identify potential confounders. The global significance of categorical variables was assessed using Wald tests and model fit was assessed using the Akaike information criterion (AIC). Likelihood-ratio tests on nested models were produced to quantify the evidence of model fit between models, and the models with the lowest AIC are reported.

## Results

### Was our sample representative?

We recruited 454 patients, 61% of all those eligible to take part. They were mostly single men of White ethnicity, unemployed and on average aged 40 years with a diagnosis of psychosis (56%) (online Table DS1). Mean NOSIE (16.0) and GAF (43.3 global functioning and 42.2 symptom severity) scores across phases were similar between the triage and routine care systems. We extracted data on the in-patient population over the same 18-month period for comparison with our sample (see Williams *et al*^7^ for the method) and found that our sample was very similar (Table DS1) to the wider population of in-patients.

In total, 484 observations from 284 different staff were collected during the study (online Table DS2), an average of 57% of eligible staff at each phase. Most were women, of non-White ethnicity, qualified nurses, aged on average 36 years. Their characteristics are what we would expect from an acute care setting in an inner-city service.

We investigated whether the characteristics of staff or patients changed over time or between systems and there were no differences for patients except that GAF symptoms and functioning increased linearly over the phases (both *P*<0.001). For staff, the triage system had a higher proportion of female staff (*P* = 0.020) and qualified staff (*P* = 0.003) and longer employment (*P* = 0.019), and over time all staff participants became significantly younger (χ^2^(1) = 6.52, *P* = 0.011). These variables were considered in sensitivity analyses when investigating differences between the two systems.

### Do patients' experiences change over time or differ between the triage and routine care systems?

Here we address questions (a) and (b). No significant difference was found in perceptions of in-patient care between the two systems (VOICE scores adjusted mean difference: 0.77, 95% CI 4.44 to 2.90, *P* = 0.68,) but perceptions deteriorated over time in both systems (Table 1). Every 6 months the mean VOICE score for the whole sample increased (coefficient: 2.72, 95% CI 1.00 to 4.43, *P*<0.005) and this model remained after adjusting for clustering and in the two sensitivity analyses (online Table DS3). There was no evidence of differences between the three ward types. There was no evidence that any factors associated with VOICE could account for the deterioration. Baseline VOICE for both systems (Table DS1) was high initially (52 in both systems) so deterioration is from an already poor view of the ward.

**Table 1:** Patient outcomes^a^

	Coefficient (95% CI)	*P*
Main outcome: Views on Inpatient Care (VOICE)^b^		
Triage system	−0.77 (−4.44 to 2.90)	0.681
Phase	2.72 (1.00 to 4.43)	0.002*
Triage system (excluding 7 participants with days until interview <7 days)	−0.54 (−4.23 to 3.16)	0.774
Triage system (adjusted for days until interview)	−0.78 (−4.45 to 2.89)	0.678

Service Satisfaction Scale: Residential services evaluation^b^		
Triage system	−1.77 (−7.07 to 3.53)	0.512
Phase	2.29 (0.15 to 4.74)	0.066
Triage system (excluding 7 participants with days until interview <7 days)	−1.53 (−6.85 to 3.79)	0.572
Triage system (adjusted for days until interview)	−1.79 (−7.09 to 3.51)	0.508

Total length of stay^c^		
Triage system	−12.35 (−37.35 to 17.86)	0.384
Phase	−16.16 (−26.84 to 5.49)	0.003*

a. Intraclass correlation coefficients (ward) were 0.06, 0.07 and 0.06 for the three outcomes respectively.

b. Linear regression covarying for time assuming a linear relationship.

c. Linear regression covarying for phase assuming a linear relationship. Standard errors and bias-corrected confidence intervals presented are from bootstrapped results using 1000 replications. Five different seeds were used and all estimates were consistent to 1 decimal place. Seven participants with <7 days from admission until interview were removed.

* *P*<0.05 after performing a sensitivity analysis of the same model with robust standard errors adjusted for clustering at ward level. Significance of all other results did not change.

No significant differences were found in satisfaction but, as with VOICE, a trend of deteriorating satisfaction was observed (*P* = 0.066). There was a trend towards worse satisfaction in the triage ward compared with the triage locality wards (mean difference 6.91, 95% CI 14.66 to 0.84, *P* = 0.08) that remained in the two sensitivity analyses. Satisfaction at the beginning of the study was not high (90.7 routine care; 84.5 triage system) so the systems are not starting from a satisfied position.

#### Does behavioural or clinical change explain deterioration in patient experience?

We found a trend of decreasing total length of stay with the average total length of stay decreasing in each successive phase by 16.16 days (*P* = 0.003, 95% bias-corrected CI 27.33 to 5.45). This finding remained in the sensitivity analysis and when adjusted for the number of in-patient care days until interview (mean difference: 11.43 days, 95% bias-corrected CI 19.39 to 4.47, *P* = 0.003). Although several factors (for example NOSIE and number of meaningful staff contacts) were associated with VOICE scores, the deterioration remained in all sensitivity analyses. In addition there was no linear trend in NOSIE change over time (χ^2^(1) = 1.34, *P* = 0.248) suggesting that this was not the cause of the change in VOICE scores over time.

### Are there differences in staff experience in the two systems of care or over time?

The same models were examined for staff but here we used time as a categorical variable (Table 2). Interactions between system and phase were significant for VOTE (χ^2^(3) = 11.03, *P* = 0.01) and for MBI (χ^2^(3) = 8.89, *P* = 0.03) but not for the IWS (χ^2^(3) = 5.80, *P* = 0.12) suggesting that there were fluctuations between phases. But, as in the patient measures, the greatest change was deterioration in staff perceptions of in-patient care over time in both triage and routine care. By phase 3 both systems were worse compared with baseline and there was no evidence of a difference between the two systems (*P* = 0.994). In the analysis of ward types only the contrast between the triage ward and triage locality wards showed a weak trend towards worse experience on the triage ward (χ^2^(1) = 2.75, *P* = 0.097).

**Table 2:** Staff primary outcomes^a^

	Coefficient (95% CI)	*P*
*Views of the Therapeutic Environment (VOTE)^b^*		
System		
Routine care system	reference	
Triage system	1.68 (−2.05 to 5.41)	0.379
Interaction: system (routine) × phase		
Baseline (routine)	reference	
Phase 1	6.58 (3.83 to 9.33)	<0.001*
Phase 2	0.25 (−3.11 to 3.16)	0.987
Phase 3	4.80 (1.30 to 8.30)	0.007*
Interaction: system (triage) × phase		
Baseline (triage)	reference	
Phase 1	0.94 (−1.81 to 3.70)	0.503
Phase 2	−0.13 (−2.90 to 2.63)	0.924
Phase 3	4.78 (1.93 to 7.63)	0.001

*Maslach Burnout Inventory Human* *Services Survey^b^*		
System		
Routine care system	reference	
Triage system	0.86 (−3.75 to 5.47)	0.715
Interaction: system (routine) × phase		
Baseline (routine)	reference	
Phase 1	5.02 (1.44 to 8.60)	0.006*
Phase 2	2.47 (−1.47 to 6.40)	0.220
Phase 3	8.61 (4.21 to 13.01)	<0.001*
Interaction: system (triage) × phase		
Baseline (triage)		
Phase 1	−0.87 (−4.60 to 2.86)	0.648
Phase 2	1.06 (−2.60 to 4.72)	0.569
Phase 3	1.76 (−1.95 to 5.46)	0.352

*Index of Work Satisfaction^b^*		
System		
Routine care system	reference	
Triage system	8.06 (0.95 to 15.16)	0.026*
Phase		
Baseline	reference	
Month 6	8.13 (3.23 to 13.05)	0.001*
Month 12	5.06 (−0.16 to 10.27)	0.057
Month 18	11.90 (6.36 to 17.43)	<0.001*

a. Intraclass correlation coefficients were 0.71, 0.64 and 0.72 for the three outcomes respectively.

b. Mixed-model linear regression including a random effect for individuals and covarying for phase (categorical, coefficients use the baseline as a comparison). A system × phase interaction was tested in each model and results are presented where this interaction was shown to be significant from a likelihood ratio test.

* *P*<0.05 after performing a sensitivity analysis of the same model with an additional level of clustering at the ward level. Significance of all other results did not change.

#### Staff burnout

Staff burnout, assessed using the MBI, shows a similar pattern of no difference between triage and routine care at baseline (mean difference: 0.86, 95% CI 3.75 to 5.47, *P* = 0.715). There is evidence of deterioration in routine care over time but relative stability in the triage system with significant differences at phase 1 (mean difference: −5.89, 95% CI −11.06 to −4.60, *P* = 0.025,) and phase 3 (mean difference: −6.85, 95% CI −12.60 to −1.10, *P* = 0.020). These results remained stable in the sensitivity analyses. Burnout was lowest on the triage locality wards.

#### Satisfaction

Results from the IWS demonstrated consistently better satisfaction in routine care staff (IWS mean difference: 8.06, 95% CI 0.95–15.16, *P*<0.05) that was mainly accounted for by much lower satisfaction in the triage locality wards (mean difference: 9.03, 95% CI 1.19 to 16.87, *P* = 0.024). As with the other staff experience measures, satisfaction deteriorated over time for both systems (mean difference: 11.90, 95% CI 6.36 to 17.43, *P*<0.001).

#### Can deterioration in staff experience be predicted?

After adjusting for variables that predict lower VOTE scores (for example shorter employment), the general deterioration in both systems remains (online Table DS4).

### What are the patient-perceived contacts in the two systems and what are their costs?

Patient-perceived meaningful contacts decreased over the study with on average an estimated change of 0.69 contacts per week (95% CI −1.12 to −0.27) for each phase, with meaningful contacts with nurses and doctors changing by −0.23 (95% CI −0.56 to 0.11) and −0.27 (95% CI −0.40 to −0.15) respectively. Despite similar levels of nurse staffing, patients in the triage system reported about half the number of meaningful one-to-one contacts with nurses (other than care coordinators) compared with those in routine care (online Table DS5). The cost of patient-perceived meaningful contacts with occupational therapists and activities was also statistically significantly lower in the triage system. The total cost of meaningful contact was estimated to be £41 lower in the triage than the routine care system in the unadjusted analysis (95% CI £7 to 75) but this estimate was highly unstable over time and between wards. The use of cluster robust standard errors increased the width of confidence intervals considerably such that differences were not statistically significant in the unadjusted comparison.

Patients on the triage ward were significantly less likely to take part in activities and have meaningful contact with other care professionals. The overall cost of reported service use was marginally lower in the triage ward compared with triage locality wards (mean difference: −£17, 95% CI −£62 to £27) but was not statistically significant and was also unstable. These data suggest that there were no substantial differences between the overall costs of reported care received in the triage ward and triage locality wards.

In summary, there were significant deteriorations in the primary outcomes (VOICE and VOTE) and no differences between the different service systems. Over the course of the study there were reductions in lengths of stay and reduced meaningful contact with staff.

## Discussion

The findings show a bleak picture of deterioration in staff and patient experience over time in all the measures in both systems and, in contrast to our expectations, the triage system of care did not benefit patient experience. Below we consider the strength of these new conclusions.

### Are there any predictors that might account for deterioration in experience?

Although the triage ward might be expected to be a more highly charged environment we found no evidence to suggest that patients viewed it less favourably than patients on the routine care wards. The deterioration in patients' perceptions and satisfaction coincided with shorter lengths of stay but not with a change in the patient population (for example NOSIE scores). It is striking that there was a substantial reduction in the average length of stay across both systems. This reflected intense managerial pressure to improve performance against static or even shrinking budgets including the cost-efficiency closure of a ward in the routine care system. As perceptions are better when patients are able to spend more time one-on-one with staff,^17^ this also becomes a bigger challenge when the ward is busier.

Nurses' perceptions and burnout did not differ significantly between the two systems, although both got worse over time, mirroring patient perceptions. There were trends for staff on triage locality wards to report lower burnout but also lower satisfaction. This may reflect a less stressful working environment with fewer numbers of acutely unwell patients but also dissatisfaction with the much slower patient turnover and frustrations over delayed discharges. No other patient or staff characteristics explained the deteriorating perceptions over time.

### Cost comparisons

The analyses suggested that the cost of meaningful staff contacts as perceived by patients might be lower in the triage system. However, the generalisability and robustness of the findings were limited. Similarly, there was no clear evidence for differences in meaningful staff contact costs between the triage ward and locality wards in the triage system. It should also be emphasised that the aim here was not to investigate overall costs, but only costs that were meaningful from the patient's perspective.

### Experience over time

A striking finding in the study is that both patients and staff reported deterioration in their experience of in-patient services regardless of where they were managed. Sadly these data chime with other reports that suggest deterioration in staff morale and patient experience working across the wider health services in recent years.^11^ This decline coincides with a period of local and national budget tightening^18,19^ and system pressure to increase efficiency that was reflected in our data by a reduction in length of admission to hospital and continuing high bed occupancy. This is likely to contribute to increased behavioural disturbance, a key predictor of patient experience. Over time there was a reduction in time spent with staff and this may be a driver of the patient experiences. But despite investigating several factors we were not able to identify specific factors that predicted deterioration.

### Strengths and limitations

One strength of our large (454 patient and 484 staff observations) 18-month long study is that it is not a mere snapshot, albeit that it is in a single National Health Service (NHS) trust. We recruited more than 50% of potential participants and we could not detect any differences between our sample and data for the whole in-patient population over the same period, although patients not recruited might have been more acutely unwell. The costs are based on data from the patient's point of view and this was a deliberate choice in line with the suggestions made in the Francis report. It does not reflect the amount of objective time spent in any staff contact. As staff in the triage system probably spend more time in administrative activities (such as community liaison) in order to resolve the patient stay within 7 days, they may not be seen by patients as spending time in direct one-to-one meaningful contacts. Reflecting the evolution of mental healthcare in the NHS we also noted changes in the wards, particularly in the routine care system. Change is usually associated with deteriorating perceptions of care as staff and patients become accustomed to the new services. This would have produced poorer perceptions in routine care compared with triage. However, we did not detect any differences suggesting that whatever effect the service change had it did not benefit triage care.

### Clinical implications

Our results indicate a pessimistic view of in-patient experience over time. We were unable to identify many predictors of deterioration except reductions in length of stay and decreased contact with staff. Services therefore need to concentrate on these characteristics as potential markers of poorer perceptions and consider ways to mitigate them perhaps by ensuring protected staff time with patients. For staff, contrary to our expectations, those in the triage ward were no more likely to report burnout than staff in routine care. Where differences appear they are mainly within the triage locality wards where staff report less burnout (as might be expected with a less acutely ill patient population) but greater dissatisfaction, possibly reflecting much slower turnover of patients and frustrations over delayed discharges or a reflection of the higher status and regard that the newer triage ward approach attracts. Setting up a triage ward system therefore requires a concentration on the non-triage elements of the system and particularly staff satisfaction, which is also likely to have an impact on patient perceptions of care.

### Future research

Future research would benefit from the inclusion of new sites and longitudinal studies tracking the impact of the introduction of interventions that may improve patient and staff perceptions of care over a period of time. Changes in care are an inevitable part of the UK NHS and we hope to explore the effects of these changes in more detail within our own rich longitudinal data-set.

In conclusion, we have discovered deterioration in patient and staff views of in-patient mental health services. This was not mitigated by the type of admission and may reflect changes to mental healthcare over the study period. This is the first time such a rigorous study pinpointing these difficulties has been carried out and it provides the baseline for future improvement.
